# Association between alcohol consumption and carotid atherosclerosis in Russia

**DOI:** 10.1038/s41598-025-16290-0

**Published:** 2025-08-27

**Authors:** Nikita A. Mitkin, Andrew N. Ryabikov, Olga A. Mirolyubova, Ulker G. Guseinova, Natalia V. Solovieva, Andrey Soloviev, Sofia Malyutina, Alexander V. Kudryavtsev

**Affiliations:** 1 Department of Community Medicine, UiT The Arctic University of Norway, N- 9037 Tromsø, Norway; 2 Northern State Medical University, Troitsky Ave., 51, Arkhangelsk, Russia 163069; 3 Novosibirsk State Medical University, Krasny Ave., 52, Novosibirsk, Russia 630091; 4 Research Institute of Internal and Preventive Medicine, Branch of Institute of Cytology and Genetics, Siberian Branch of the Russian Academy of Sciences, Bogatkova st., 175/1, Novosibirsk, Russia 630089

**Keywords:** Alcohol, Carotid, Atherosclerosis, Plaque, Intima media, Stenosis, Alcohol use disorder, Atherosclerosis, Risk factors, Addiction

## Abstract

The relationship between alcohol consumption levels and carotid atherosclerosis remains controversial. We investigated this association in 2357 general population participants and 272 in-patients treated for alcohol-related disorders (narcology patients) aged 35–69 years in Arkhangelsk, Russia (Know Your Heart study, 2015–2017). Participants were categorized as non-drinking, non-problem drinking, hazardous drinking, harmful drinking, and narcology patients. Carotid atherosclerosis was assessed via carotid intima-media thickness (CIMT), plaque score (0–6 points), and presence of stenosis ≥ 50%. We used quantile regression for CIMT, linear regression for plaque score, and logistic regression for stenosis. Models were adjusted for sociodemographic factors and dysmetabolic conditions (abdominal obesity, dyslipidemia, hypertension, diabetes, and systemic inflammation). Compared to non-problem drinking, narcology patients had significantly higher CIMT (+ 0.05 mm), plaque score (+ 0.57 points), and odds of stenosis (OR = 2.10) independent of dysmetabolic conditions. Hazardous drinking participants had higher CIMT (+ 0.03 mm) and plaque score (+ 0.15 points), which became non-significant after adjusting for hypertension. Harmful drinking participants did not differ in atherosclerosis markers from those with non-problem drinking. Non-drinking participants had higher CIMT (+ 0.02 mm) and plaque score (+ 0.30 points). These findings in a middle-aged Russian population suggest that chronic severe alcohol consumption can affect carotid artery walls directly, while hazardous drinking is linked to atherosclerosis through hypertensive mechanisms.

**Supplementary Information:**

The online version contains supplementary material available at 10.1038/s41598-025-16290-0.

## Introduction

Atherosclerosis is a chronic inflammatory and dysmetabolic disease characterized by plaque buildup in arterial walls. It is the primary cause of cardiovascular diseases, which accounts for 32% (18 million) of global deaths annually^1,2^. While dyslipidemia, hypertension, diabetes, and smoking are well-studied risk factors for atherosclerosis, the role of alcohol consumption remains incompletely understood^3^.

Previous studies show a non-linear association between alcohol consumption and cardiovascular risk. Moderate drinking can be associated with a 20–36% reduction in cardiovascular mortality compared to abstinence across various settings and populations^4–11^. This protective effect may be due to increased high-density lipoprotein levels and reduced inflammatory markers. Other potential mechanisms may include improved lipid fraction profiles, antioxidant effects, enhanced endothelial function, better insulin sensitivity, and plaque stabilization^12–15^. However, these benefits appear to be dose-dependent, as heavy drinking was described as significantly increasing cardiovascular mortality due to oxidative stress, accelerated smooth muscle cell proliferation, and weakened endothelial repair^16–18^. Alcohol can also affect inflammatory pathways, arterial stiffness, and mitochondrial function^19–21^.

The threshold between potentially beneficial and harmful effects of alcohol consumption on cardiovascular system remains poorly defined, particularly in populations with distinct drinking patterns^22,23^. The harmful effects are most experienced by people with alcohol use disorders, who may have more than twice the cardiovascular mortality rate in the general population^24–26^. However, the degree of atherosclerotic vascular damage depending on the levels of alcohol consumption remains understudied, particularly in non-Western regions^27^. This knowledge gap is particularly critical in populations with a high burden of subclinical atherosclerosis. This applies to Russia where the atherosclerosis prevalence has been reported as high as 73.6% in adults aged 40–67 years^28^.

Russia also has a wide diversity of alcohol consumption patterns and represents a unique environment for studying its health outcomes. The country’s cardiovascular mortality rate exceeds the European average by 12%, while the prevalence of alcohol use disorders and dependence is two to three times higher than in European countries^29,30^. Thus, we aimed to assess markers of carotid atherosclerosis in the general Russian population aged 35–69 years stratified by levels of alcohol consumption and in individuals treated for alcohol-related disorders.

## Methods

### Study design and participants

We used data from the Know Your Heart (KYH) cross-sectional study (2015–2017), part of the International Project on Cardiovascular Disease in Russia. The study population included 2357 men and women aged 35–69 from the general population of Arkhangelsk (main study), and 275 in-patients treated for alcohol-related diagnoses (narcology patients). The details of KYH have been described elsewhere^31^.

Participants in the main study were recruited as a random sample from the database of the regional health insurance fund, stratified by age, sex, and city district. Recruitment involved trained interviewers visiting randomly selected addresses to identify eligible participants. The response rate for the main study was 68%. Narcology patients were recruited from in-patients of the Arkhangelsk Clinical Psychiatric Hospital. Eligible patients had a documented history of at least five years of alcohol abuse and had been in treatment for at least one week after hospitalization. The response rate for this group was 85%.

### Data collection

Data collection included a baseline interview and a health check. Trained interviewers conducted baseline interviews at participants’ homes for the main study and at the hospital for narcology patients using Computer-Assisted Personal Interviews. They collected information on demographics, socio-economic status, and health behaviors, with a focus on alcohol consumption over the past 12 months. The CAGE^32^ was used to screen for alcohol dependence. Baseline interviews also included questions about signs of harmful drinking: frequency of “zapoi” episodes (an alcohol drinking period of two or more days, during which a person falls out of a routine social life), hangover, excessive drunkenness, sleeping in clothes at night due to drunkenness, and failing to fulfill family or other obligations because of being drunk.

The health check was performed by trained medical staff at the polyclinic of Northern State Medical University in Arkhangelsk and consisted of a medical interview, physical examination, and blood sampling. Medical interview covered participants’ medical history, health-related behaviors, and included the second round of collecting alcohol-drinking data using AUDIT^33^. Physical examination involved anthropometric measurements. Height (cm) was measured using a Seca® 217 stadiometer (Seca Limited), weight (kg) – using a TANITA BC 418 body composition analyzer (TANITA, Europe GmbH), waist circumference (WC) was measured with the Seca®201 tape (Seca Limited). Height and WC were measured twice, and the average of the two measurements was used in the analysis. Systolic and diastolic blood pressure (SBP and DBP) were measured at the brachial artery using an OMRON 705 IT automatic tonometer (OMRON Healthcare). Both SBP and DBP were measured three times after five minutes of rest. The mean values of the second and third measurements were used for analysis. Vascular ultrasound examination of carotid arteries was performed using a 6–13 MHz linear transducer (Vivid q, GE Healthcare) in ECG gated manner following a standardized protocol. Ultrasound images were analyzed offline using EchoPAC software (v.113, GE-Vingmed AS), with a common carotid intima-media thickness and carotid plaques evaluated according to the Mannheim Consensus. Two investigators from Reading Lab of the KYH project (Novosibirsk, Russia), performed off-line vascular analysis, with intra- and inter-reader variability regularly assessed.

Blood samples were collected after at least four hours of fasting, centrifuged, aliquoted, frozen at − 80 °C, and transported to Lytech laboratory in Moscow on dry ice. All laboratory analyses were performed in one batch after data collection was completed. Laboratory personnel were blind to participants’ characteristics. Total cholesterol, high-density lipoprotein cholesterol (HDL), low-density lipoprotein cholesterol (LDL), and triglycerides levels were assessed in serum by enzyme calorimetry, glycated hemoglobin (HbA1c) and high-sensitivity C-reactive protein (hs-CRP) – by immunoturbidimetric tests (AU 680; Chemistry System Beckman Coulter).

### Exposure

Participants were categorized into five levels of alcohol consumption based on their drinking characteristics:

1. Non-drinking: No alcohol consumption in the past 12 months reported.
2. Non-problem drinking: Alcohol consumption in the past 12 months with AUDIT scores < 8 and/or CAGE scores < 2.
3. Hazardous drinking: Alcohol consumption in the past 12 months with AUDIT scores of 8–15 and/or CAGE scores of 2–3.
4. Harmful drinking: Alcohol consumption in the past 12 months with at least one of the following: a) AUDIT score ≥ 16^33,34^, (b) CAGE score of 4^32,35^, (c) ≥ 1 episodes of “zapoi” in the past year and/or reporting hangover and/or excessive drunkenness and/or sleeping in clothes at night because of drunkenness and/or failing to fulfill family or social obligations because of alcohol ≥ 2 times per week.
5. Narcology patients: Individuals currently receiving inpatient treatment for alcohol-related diagnoses.

The validity of this approach has been demonstrated in earlier studies by showing differences between groups in annual alcohol consumption, seeking for medical care for alcohol-related problems, and blood biomarkers of excessive drinking (GGT and CDT)^24,36–38^.

### Outcome

To assess early signs of atherosclerosis, we analyzed the average carotid intima media thickness (CIMT, mm) on the far wall of the distal common carotid artery. Measurements were made on plaque-free segment and then averaged over 3–5 cardiac cycles on both the left and right sides, with the higher value selected for analysis.

To evaluate the burden of carotid plaques, we used a plaque score, assigning a value of one for the presence of one or more plaques in one of the six carotid segments (common carotid artery, bifurcation, and internal carotid artery on both sides). Accordingly, the maximum individual score was six. This tool was used in the KYH study earlier by Imahori et al.^39^.

To assess advanced carotid atherosclerosis, we analyzed the presence of hemodynamically significant carotid stenosis defined as a ≥ 50% lumen narrowing in at least one segment of the internal or common carotid arteries on either side (later referred to as carotid stenosis). The degree of stenosis was calculated using planimetry from transverse ultrasound images.

### Covariates

We controlled for potential confounders which included age (years), higher education (yes/no), marital status (living with a spouse or partner/no), and current smoking status (yes/no). Abdominal obesity, hypertension, diabetes mellitus, dyslipidemia and systemic inflammation were assessed as potential mediators.

Abdominal obesity was defined as a waist-to-height ratio of ≥ 0.5. Diabetes was identified as glycated hemoglobin (HbA1c) levels of ≥ 6.5%, self-reported use of antidiabetic medications, or a self-reported diagnosis of diabetes, with the specification of the type of diabetes and the prescribed treatment (insulin, drugs, or diet). Hypertension was defined as SBP ≥ 140 mm Hg, DBP ≥ 90 mm Hg, or self-reported use of antihypertensive medications. Dyslipidemia was defined as total cholesterol ≥ 5.2 mmol/L, triglycerides > 1.7 mmol/L and/or LDL-cholesterol > 3.0 mmol/L and/or HDL-cholesterol < 1.0 mmol/L for men or < 1.2 mmol/L for women and/or self-reported use of lipid-lowering medications. Systemic inflammation was defined by hs-CRP levels between 2 and 10 mg/L. Non-HDL cholesterol (non-HDL) was calculated as the difference between total and HDL-cholesterol.

### Statistical analysis

We used counts and percentages (%) to describe categorical variables, and either means with standard deviations (M ± SD) or medians with interquartile ranges (Me [Q1, Q3]) for continuous variables, depending on their distribution. Trends were assessed using the Jonckheere–Terpstra test for skewed continuous data, the Cochran–Armitage test for binary variables, and the linear-by-linear association test for normally distributed continuous variables.

We used quantile (median) regression to assess the association between alcohol consumption level and the CIMT. Linear regression was used to assess the association with plaque score (0–6 points), logistic regression – with the presence of carotid stenosis (0/1). To explore potential interaction effects between alcohol consumption levels and sex, we compared models with and without interaction terms by using likelihood-ratio test, which revealed no significant sex differences. Therefore, the analyses were performed without stratification by sex. We built regression models in a stepwise manner. Model 1 involved adjusted for sex, age, education, marital status, and smoking. In Models 2a-2e, we by turn performed further adjustments for abdominal obesity, dyslipidemia, hypertension, diabetes, and systemic inflammation, respectively. Model 3 was adjusted for all these conditions together. Results from linear and quantile regression models were presented as adjusted B-coefficients with 95% confidence intervals (CI), which reflected corresponding mean and median differences, while adjusted odds ratios (OR) with 95% CIs were reported for logistic regression.

In sensitivity analyses (Supplementary Table 1), we repeatedly tested the associations between alcohol consumption and CIMT in participants without plaques and with the presence of stenosis in participants with plaques. Supplementary Table 2 details the fully adjusted models (Model 3) for CIMT, plaque score, and carotid stenosis, presenting the regression coefficients for both alcohol exposure and all model covariates.

All analyses were performed using Stata 18.0 (StataCorp, College Station, Texas, USA).

### Ethical approval

This study was conducted in line with the principles of the Declaration of Helsinki and other relevant guidelines and regulations. The Know Your Heart study was approved by the ethics committees of the London School of Hygiene and Tropical Medicine (approval number 8808; received 24 February 2015), Novosibirsk State Medical University (approval number 75; received 21 May 2015), the Research Institute of Internal and Preventative Medicine, Novosibirsk (no approval number; received 26 December 2014), and the Northern State Medical University, Arkhangelsk (approval number 01/01–15; received 27 January 2015). Recruitment of the narcological sub-study participants was approved by Research Ethics Committee at Northern State Medical University, Arkhangelsk, Russia (Protocol No 05/11–16, received 02/11/2016). All participants signed an informed consent form.

## Results

### Socio-demographic and health parameters

Non-problem drinking participants were the largest group (N = 1,656), followed by hazardous drinking (N = 360), narcology patients (N = 272), non-drinking (N = 236), and harmful drinking (N = 105) groups (Table 1). Narcology patients were the youngest group (median 47.9 years), and non-drinking participants were the oldest (median 57.6 years). Proportion of men increased from non-problem drinking (30.2%) to hazardous drinking (78.1%), harmful drinking (87.6%), and narcology patients (76.1%). Higher education was most common in non-problem drinking group (39.3%) and least common in narcology patients (9.6%). Marriage proportions were lower in narcology patients (20.7%) compared to other groups (53.3–67.8%).

**Table 1:** Socio-demographic and health characteristics of participants by levels of alcohol consumption.

Characteristics	Levels of alcohol consumption					*p*-trend
	Non-drinking	Non-problem	Hazardous	Harmful	Narcology	
	(N = 236)	(N = 1,656)	(N = 360)	(N = 105)	(N = 272)	
Age, Me (Q1; Q3)	57.6 (48.7; 64.9)	54.5 (45.6; 62.7)	50.7 (44.0; 59.1)	51.7 (44.3; 59.1)	47.9 (41.0; 54.7)	< 0.001^a^
Male sex, N (%)	109 (46.2)	500 (30.2)	281 (78.1)	92 (87.6)	207 (76.1)	< 0.001^b^
Higher education, N (%)	64 (27.1)	651 (39.3)	120 (33.3)	19 (18.1)	26 (9.6)	< 0.001^b^
Married, N (%)	143 (60.6)	1,015 (61.3)	244 (67.8)	56 (53.3)	56 (20.7)	< 0.001^b^
Current smoker, N (%)	52 (22.0)	290 (17.5)	138 (38.3)	68 (64.8)	223 (82.0)	< 0.001^b^
WHtR, M (SD)	0.55 (0.09)	0.54 (0.09)	0.55 (0.07)	0.53 (0.08)	0.50 (0.06)	< 0.001^c^
Self-reported diabetes, N (%)	20 (8.5)	121 (7.3)	19 (5.3)	6 (5.7)	6 (2.2)	< 0.001^b^
Use of antidiabetics, N (%)	15 (6.4)	105 (6.3)	15 (4.2)	4 (3.8)	4 (1.5)	< 0.001^b^
HbA1C (%), Me (Q1; Q3)	5.47 (5.21; 5.76)	5.47 (5.20; 5.75)	5.43 (5.18; 5.73)	5.31 (5.10; 5.59)	5.38 (5.18; 5.68)	0.004^a^
SBP, mm Hg, M (SD)	131.3 (21.9)	131.1 (19.9)	136.9 (19.4)	135.7 (20.4)	124.8 (17.9)	0.021^c^
DBP, mm Hg, M (SD)	81.1 (11.9)	82.6 (11.2)	87.6 (11.8)	86.1 (12.1)	82.7 (11.5)	< 0.001^c^
Use of antihypertensives, N (%)	109 (46.2)	686 (41.4)	124 (34.4)	34 (32.4)	44 (16.2)	< 0.001^b^
Total cholesterol, mmol/L, M (SD)	5.31 (1.15)	5.45 (1.09)	5.45 (1.20)	5.14 (0.91)	4.90 (1.01)	< 0.001 ^c^
TG, mmol/L, Me (Q1; Q3)	1.16 (0.80; 1.76)	1.19 (0.85; 1.75)	1.33 (0.90; 1.90)	1.21 (0.88; 1.73)	1.31 (1.04; 1.67)	< 0.001^a^
LDL, mmol/L, M (SD)	3.65 (0.95)	3.67 (0.88)	3.70 (0.99)	3.40 (0.77)	3.23 (0.81)	< 0.001^c^
HDL, mmol/L, M (SD)	1.38 (0.38)	1.47 (0.35)	1.41 (0.39)	1.48 (0.46)	1.40 (0.35)	0.184^c^
Non-HDL, mmol/L, M (SD)	3.94 (1.10)	3.98 (1.07)	4.04 (1.19)	3.66 (0.92)	3.49 (0.91)	< 0.001^c^
Use of statins, N (%)	34 (14.4)	163 (9.8)	31 (8.6)	8 (7.6)	2 (0.7)	< 0.001^b^
Hs-CRP, mg/L, Me (Q1; Q3)	1.45 (0.63; 3.35)	1.58 (0.69; 3.47)	1.72 (0.73; 3.82)	1.73 (0.89; 4.32)	2.96 (1.36; 7.06)	< 0.001^a^

*P*-values for trends from: (a) Jonckheere–Terpstra test, (b) Cochran-Armitage test, and (c) linear-by-linear test. Definitions: WHtR; waist-to-height ratio, HbA1C; glycated hemoglobin A1, SBP; systolic blood pressure, DBP; diastolic blood pressure, TG; triglycerides, LDL; low density lipoprotein, HDL; high density lipoprotein, non-HDL; non-HDL cholesterol, Hs-CRP; high-sensitive C-reactive protein.

Smoking prevalence increased from non-problem drinking participants (17.5%) to narcology patients (82.0%). Blood pressure was highest in hazardous and harmful drinking groups (mean SBP 136.9 and 135.7 mmHg; mean DBP 87.6 and 86.1 mmHg) and lowest in narcology patients (mean SBP 124.8 mm Hg; mean DBP 82.7 Hg mm). Use of antihypertensive was highest in non-drinking participants (46.2%) and lowest in narcology patients (16.2%). Waist-to-height ratio was lowest in narcology patients (mean 0.50) compared to other groups (means 0.53–0.55). Both self-reported diabetes and antidiabetics use were highest in non-drinking group (8.5% and 6.4%) and lowest in narcology patients (2.2% and 1.5%). Similarly, median levels of HbA1c were highest in non-drinking and non-problem drinking groups (5.47% in both) and lowest in narcology patients (5.38%).

Total cholesterol, LDL, and non-HDL cholesterol levels were lowest in narcology patients (means 4.90, 3.23, and 3.49 mmol/L, respectively) and highest in the hazardous drinking group (means 5.45, 3.70, and 4.04 mmol/L, respectively). Triglycerides were elevated in hazardous drinking and narcology patients (medians 1.33 and 1.31 mmol/L). Statin use was highest in non-drinking group (14.4%) and lowest in narcology patients (0.7%). Hs-CRP levels were notably higher in narcology patients (median 2.96 mg/L) compared to other groups (medians 1.45–1.73 mg/L).

Except for HDL, all the analyzed socio-demographic and health characteristics showed statistically significant trends (p < 0.05) relative to the alcohol consumption level.

### Alcohol consumption levels and carotid atherosclerosis

In the unadjusted analyses, carotid intima media thickness showed little variation across drinking categories (Table 2). The averaged maximum values for either right or left arteries ranged from median 0.85 mm (non-problem drinking) to 0.91 mm (non-drinking), with no significant trend (*p* = 0.535). Similar patterns were observed for the right artery (medians 0.79–0.84 mm, *p* = 0.946) and the left artery (medians 0.81–0.86 mm, *p* = 0.358) taken separately.

**Table 2:** Characteristics of carotid atherosclerosis by levels of alcohol consumption.

Characteristics	Levels of alcohol consumption					*p*-trend
	Non-drinking	Non-problem	Hazardous	Harmful	Narcology	
CIMT max of both sides (mm), Me (Q1; Q3)	0.91 (0.79; 1.06)	0.85 (0.73; 0.98)	0.89 (0.76; 1.02)	0.88 (0.80; 1.00)	0.86 (0.76; 0.98)	0.535^a^
CIMT Max Right (mm), Me (Q1; Q3)	0.84 (0.72; 0.99)	0.79 (0.68; 0.91)	0.83 (0.70; 0.93)	0.80 (0.71; 0.91)	0.81 (0.71; 0.91)	0.946^a^
CIMT Max Left (mm), Me (Q1; Q3)	0.86 (0.75; 0.97)	0.81 (0.70; 0.93)	0.84 (0.72; 0.97)	0.85 (0.74; 0.95)	0.83 (0.73; 0.95)	0.358^a^
Plaque score (points), Me (Q1; Q3)	2 (0; 3)	1 (0; 2)	1 (0.; 3)	2 (1; 3)	2 (0; 3)	< 0.001^a^
*Plaques, N (%)*						
At least one location	168 (71.2)	928 (56.0)	238 (66.1)	80 (76.2)	192 (70.6)	< 0.001^b^
BCA, right	118 (50.0)	624 (37.7)	167 (46.4)	51 (48.6)	122 (44.9)	0.010^b^
BCA, left	127 (53.8)	638 (38.5)	171 (47.5)	59 (56.2)	146 (53.7)	< 0.001^b^
CCA, right	27 (11.4)	99 (6.0)	23 (6.4)	12 (11.4)	20 (7.4)	0.952^b^
CCA, left	27 (11.4)	138 (8.3)	36 (10.0)	18 (17.1)	48 (17.6)	< 0.001^b^
ICA, right	68 (28.8)	286 (17.3)	90 (25.0)	26 (24.8)	95 (34.9)	< 0.001^b^
ICA, left	61 (25.8)	260 (15.7)	93 (25.8)	32 (30.5)	103 (37.9)	< 0.001^b^
*Stenosis, N (%)*						
At least one location	26 (11.0)	101 (6.1)	31 (8.6)	16 (15.2)	29 (10.7)	0.015^b^
CCA, right	10 (4.2)	39 (2.4)	18 (5.0)	6 (5.7)	6 (2.2)	0.711^b^
CCA, left	12 (5.1)	42 (2.5)	10 (2.8)	8 (7.6)	14 (5.1)	0.057^b^
ICA, right	13 (5.5)	38 (2.3)	11 (3.1)	4 (3.8)	13 (4.8)	0.261^b^
ICA, left	8 (3.4)	36 (2.2)	7 (1.9)	8 (7.6)	19 (7.0)	< 0.001^b^

*P*-values for trends from: (a) Jonckheere–Terpstra test, (b) Cochran-Armitage test. Definitions: CIMT; intima media thickness in common carotid artery, BCA; bifurcation of carotid artery, CCA; common carotid artery, ICA; internal carotid artery.

The proportion of having at least one plaque was significantly higher in harmful drinking (76.2%), non-drinking (71.2%) groups, and narcology patients (70.6%) compared to non-problem drinking participants (56.0%, *p* < 0.001). This pattern was reflected in the plaque scale scores, with non-problem drinking category showing the lowest burden (median 1 point) compared to other groups (median 2 points, *p* for trend < 0.001). Bifurcation plaques were most frequent in harmful drinkers (right: 48.6%, left: 56.2%) and non-drinkers (right: 50.0%, left: 53.8%) compared to non-problem drinkers (right: 37.7%; left: 38.5%). Internal carotid artery plaques showed highest prevalence in narcology patients (right: 34.9%, left: 37.9%) compared to non-problem drinking participants (right: 17.3%, left: 15.7%). Common carotid plaques demonstrated a significant trend only on the left side, with highest prevalence in narcology patients (17.6%) and harmful drinking (17.1%) versus non-problem drinking group (8.3%).

Overall, the prevalence of carotid stenosis was highest in harmful drinking (15.2%), followed by non-drinking group (11.0%) and narcology patients (10.7%), and lowest in non-problem drinking participants (6.1%, p = 0.015). Stenosis of left internal carotid artery was most frequent in harmful drinking (7.6%) and narcology patients (7.0%), and least frequent in non-problem drinking (2.2%). Right internal carotid stenosis showed no significant trend (*p* = 0.261). Left common carotid stenosis demonstrated a borderline significant trend (*p* = 0.057), with highest prevalence in harmful drinking group (7.6%).

In multivariable regression analyses, CIMT in hazardous drinking and narcology patients had significantly higher median values (excess of + 0.03 mm and + 0.05 mm, respectively) compared to non-problem drinking participants with adjustments for sex, age, education, marital status and smoking (Model 1) (Table 3). For hazardous drinking, this association strengthened when adjusted for dyslipidemia and hypertension (+ 0.04 mm in Models 2b and 2c), while maintaining + 0.03 mm in remaining models. Narcology patients consistently demonstrated a + 0.05 mm median difference across models, with a minor reduction to + 0.04 mm only when adjusted for systemic inflammation (Model 2e). Harmful drinking category had no significant differences in median CIMT across models, showing only an insignificant increase to + 0.02 mm with hypertension adjustment. In the non-drinking group, the median CIMT difference increased from a non-significant + 0.02 mm in Model 1 to significant + 0.03 mm with adjustment for hypertension and was significant (+ 0.02 mm) in the fully adjusted model.

**Table 3:** Regression analyses of the associations between alcohol consumption levels and carotid atherosclerosis characteristics.

Outcomes and Drinking Groups	Model 1	Model 2a	Model 2b	Model 2c	Model 2d	Model 2e	Model 3
Carotid Intima Media Thickness (mm), quantile regressions, B (95%CI)							
Non-drinking	0.02 (− 0.01; 0.04)	0.01 (− 0.01; 0.04)	0.02 (− 0.00; 0.04)	**0.03 (0.01; 0.05)**	0.02 (− 0.00; 0.05)	0.02 (− 0.01; 0.04)	**0.02 (0.00; 0.04)**
Non- problem	Ref	Ref	Ref	Ref	Ref	Ref	Ref
Hazardous	**0.03 (0.01; 0.05)**	**0.03 (0.01; 0.05)**	**0.04 (0.02; 0.05)**	**0.04 (0.02; 0.05)**	**0.03 (0.01; 0.05)**	**0.03 (0.02; 0.05)**	**0.03 (0.01; 0.05)**
Harmful	− 0.00 (− 0.04; 0.03)	− 0.00 (− 0.04; 0.04)	0.01 (− 0.03; 0.04)	0.02 (− 0.02; 0.05)	− 0.00 (− 0.04; 0.03)	0.00 (− 0.03; 0.03)	− 0.00 (− 0.03; 0.03)
Narcology	**0.05 (0.03; 0.07)**	**0.05 (0.02; 0.07)**	**0.05 (0.03; 0.08)**	**0.05 (0.02; 0.08)**	**0.05 (0.03; 0.08)**	**0.04 (0.02; 0.07)**	**0.05 (0.03; 0.08)**
*Plaque score (points), linear regressions, B (95%CI)*							
Non-drinking	**0.28 (0.09; 0.47)**	**0.28 (0.09; 0.47)**	**0.28 (0.09; 0.47)**	**0.30 (0.11; 0.49)**	**0.28 (0.09; 0.47)**	**0.28 (0.09; 0.47)**	**0.30 (0.11; 0.49)**
Non-problem	Ref	Ref	Ref	Ref	Ref	Ref	Ref
Hazardous	**0.18 (0.02; 0.34)**	**0.17 (0.01; 0.33)**	**0.18 (0.02; 0.34)**	0.14 (− 0.02; 0.30)	**0.18 (0.02; 0.34)**	**0.18 (0.02; 0.34)**	0.15 (− 0.01; 0.31)
Harmful	0.20 (− 0.10; 0.50)	0.20 (− 0.10; 0.50)	0.21 (− 0.09; 0.51)	0.19 (− 0.10; 0.48)	0.20 (− 0.10; 0.50)	0.20 (− 0.10; 0.50)	0.20 (− 0.09; 0.49)
Narcology	**0.53 (0.31; 0.75)**	**0.54 (0.32; 0.76)**	**0.54 (0.32; 0.76)**	**0.55 (0.33; 0.76)**	**0.51 (0.29; 0.73)**	**0.52 (0.30; 0.75)**	**0.57 (0.35; 0.79)**
*Presence of Stenosis, logistic regressions, OR (95%CI)*							
Non-drinking	1.47 (0.90; 2.42)	1.47 (0.89; 2.42)	1.47 (0.89; 2.43)	1.52 (0.92; 2.51)	1.48 (0.90; 2.44)	1.49 (0.91; 2.46)	1.54 (0.93; 2.54)
Non-problem	Ref	Ref	Ref	Ref	Ref	Ref	Ref
Hazardous	1.21 (0.76; 1.93)	1.21 (0.76; 1.94)	1.21 (0.76; 1.93)	1.11 (0.69; 1.78)	1.22 (0.76; 1.94)	1.21 (0.75; 1.93)	1.14 (0.71; 1.84)
Harmful	1.83 (0.96; 3.48)	1.83 (0.96; 3.48)	1.85 (0.97; 3.51)	1.84 (0.98; 3.44)	1.84 (0.97; 3.50)	1.83 (0.96; 3.47)	1.85 (0.98; 3.48)
Narcology	**1.80 (1.01; 3.19)**	**1.79 (1.00; 3.20)**	**1.93 (1.09; 3.42)**	**2.04 (1.15; 3.62)**	**1.83 (1.02; 3.26)**	**1.79 (1.01; 3.19)**	**2.10 (1.18; 3.76)**

Significant associations (*p* < 0.05) are highlighted in bold. Model 1: adjusted for sex, age, higher education, marital status, smoking; Model 2a: adjusted as Model 1 plus abdominal obesity; Model 2b: adjusted as Model 1 plus dyslipidemia; Model 2c: adjusted as Model 1 plus hypertension; Model 2d: adjusted as Model 1 plus diabetes; Model 2e: adjusted as Model 1 plus systemic inflammation; Model 3: adjusted as Model 1 plus abdominal obesity, dyslipidemia, hypertension, diabetes, systemic inflammation.

As for the plaque score, narcology patients had a rise in the mean value of + 0.53 points in Model 1 and of + 0.57 points in the fully adjusted model relative to non-problem drinking participants. This association reduced slightly with adjustment for diabetes (+ 0.51 points) but increased with adjustment for hypertension (+ 0.55 points). Hazardous drinking category demonstrated a significant mean difference of + 0.18 points in Model 1, which decreased to non-significant difference of + 0.14 points with adjustment for hypertension and + 0.15 points in the fully adjusted model. Harmful drinking group showed consistent but non-significant elevations of approximately + 0.20 points across all models, being between hazardous drinking and narcology patients in point estimates of the differences. Non-drinking group maintained consistently elevated mean plaque scores of + 0.28 points across most models, increasing slightly to + 0.30 points in Models 2c and 3 compared to non-problem drinkers.

Regarding stenosis, narcology patients had an increase in odds ratios from 1.80 in Model 1 to 2.10 in the fully adjusted model compared to non-problem drinking group. This association was strengthened with adjustments for dyslipidemia (OR = 1.93) and hypertension (OR = 2.04). Hazardous drinking category showed no significant differences compared to non-problem drinking, with lowest odds ratio values in the model adjusted for hypertension and in the fully adjusted model (ORs = 1.11 and 1.14, respectively, versus 1.21 in other models). Harmful drinking maintained consistently elevated but non-significant ORs of 1.83–1.85 across all models, with point estimates being close to those for narcology patients. Non-drinking category had an increase in non-significant OR estimates from 1.47 in Model 1 to 1.54 in the fully adjusted model, peaking with hypertension adjustment (OR = 1.52).

### Sensitivity analysis

In a subsample of participants (N = 1,023, 38.9% of the total sample) without plaques, narcology patients maintained elevated median CIMT values (+ 0.04 to + 0.06 mm across models) with the strongest association in the fully adjusted model (+ 0.06 mm). However, adjustment for systemic inflammation attenuated this association (+ 0.03 mm). Hazardous drinking showed modest CIMT increases (+ 0.03 to + 0.04 mm) which were significant in Model 1 with basic adjustment and in Model 2b with dyslipidemia entered, indicating possible mediating effects of other analyzed health conditions. The harmful drinking category demonstrated a significant association only with adjustment for hypertension (+ 0.02 mm), while non-drinkers showed no significant associations, contrasting with the main analysis.

The associations of alcohol consumption levels with carotid stenosis in a subsample of participants with plaques (N = 1,606, 61,1% of the total sample) were weaker compared to the main findings. The significant associations observed for narcology patients in the main analysis (OR range: 1.80–2.10) were reduced and became non-significant (OR range: 1.46–1.67).

## Discussion

This study assessed the manifestations of carotid atherosclerosis in relation to the levels of alcohol consumption in a Russian adult population sample with addition of patients treated for alcohol-related disorders (narcology patients). Using stepwise adjustment for dysmetabolic conditions such as dyslipidemia, hypertension, diabetes, abdominal obesity, and systemic inflammation, we also looked to explore potential pathways linking alcohol intake to atherosclerotic changes.

The central finding was the increased atherosclerotic burden in narcology patients. Narcology patients had significantly higher carotid intima media thickness, plaque burden, and odds of arterial stenosis compared to non-problem drinkers, regardless of the adjustments for the key cardiovascular risk factors and systemic inflammation. Notably, these associations strengthened after adjusting for dyslipidemia and hypertension, suggesting these conditions act as negative confounders rather than mediators. This aligns with our observation that narcology patients had lower systolic blood pressure and prevalence of dyslipidemia, diabetes and antihypertensive medication use compared to other groups. The strengthening of associations after adjustment suggests that the direct detrimental effect of chronic severe drinking on atherosclerosis was partially masked by the lower prevalence of dysmetabolic conditions in this group.

The lipid profile observed in the narcology group (lower LDL despite elevated triglycerides) warrants special attention. This paradoxical finding is consistent with recent evidence from Suzuki et al. who demonstrated that alcohol cessation was significantly associated with increased LDL-C^40^. Notably, we observed that non-HDL cholesterol, a comprehensive marker of atherogenic particles, was also lowest in this group. Therefore, our data reflects the LDL-suppressing effect of heavy drinking. However, this potential benefit is likely negated by other pro-atherogenic factors, such as hypertriglyceridemia and systemic inflammation. This suggests triglycerides and hs-CRP are the telling indicators of cardiovascular risk in heavy drinkers, rather than LDL and non-HDL.

These results extend limited research on atherosclerosis markers in patients with alcohol use disorder. Previously, Senat et al. showed elevated atherogenic plasma indices and oxidative stress markers in AUD patients^41^, while neuroimaging study by Durazzo et al. demonstrated higher vascular and neuronal damage from impaired perfusion and inflammation^42^. The markedly higher hs-CRP levels we observed in narcology patients supports that chronic inflammation may mediate alcohol’s effects on vascular health. The pathophysiological mechanism likely involves alcohol metabolism generating reactive oxygen species that damage endothelial cells, triggering inflammatory cascades that serve as a ground for atherogenesis^16–18^. Additionally, despite lower total cholesterol, LDL and non-HDL cholesterol levels in narcology patients, their elevated triglycerides support alcohol-related dysregulation of lipid metabolism contributing to atherogenesis^43,44^. The latter cab be supported by the results of the Mendelian Randomization study on the causal effect of triglycerides on the development of coronary heart disease, one of the major atherosclerosis outcomes^45^. The influence of high triglyceride levels on the development of coronary heart disease was also found in a cohort study with a Russian population sample^46^.

Participants with hazardous drinking also showed differences from non-problem drinking group in atherosclerotic manifestations. Their higher CIMT values became even more increased after adjustments for dyslipidemia and hypertension. However, the difference in plaque score decreased following the adjustment for hypertension. This indication of a mediation effect aligns with the cardiovascular risk profile of hazardous drinkers, who had the highest blood pressure levels and elevated pro-atherogenic lipid fractions among all groups. The attenuation of the plaque score difference after the adjustment hypertension indicates that hazardous drinking likely leads to atherosclerosis through its hypertensive effects rather than direct vascular injury. This can be explained by established mechanisms of alcohol-induced hypertension, including increased sympathetic activity^47^, activation of the renin–angiotensin–aldosterone system^48^, and impaired vasodilatory responses due to reduced nitric oxide bioavailability^49^. However, such explanation was not applicable to the group of narcology patients, where atherosclerotic changes appeared independent of traditional risk factors.

Our findings both support and challenge previous research. For instance, Britton et al. showed that heavy drinkers (112 + grams of ethanol for women, and 168 + grams for men per week) had higher CIMT compared to moderate drinkers in two British cohorts^50^. Similarly, a large-scale Chinese study found that higher alcohol intake was associated with increased carotid plaque number and size, supporting a potential causal relationship between alcohol and atherosclerosis^51^. However, a comparable Russian study found no such association but identified links between carotid wall angiopathy and binge drinking^52^. In a European multi-center study, Laguzzi et al. found that heavy alcohol consumption did not significantly increase CIMT and even showed protective associations with its 30-month progression^53^. The variation in findings and effect sizes across these studies likely reflects differences in alcohol exposure definitions, population risk profiles, and economic and cultural contexts.

Harmful drinking group showed no significant differences in atherosclerosis manifestations compared to non-problem drinking participants, although their point estimates of the associations consistently fell between hazardous drinkers and narcology patients. This finding contrasts with previous research demonstrating positive associations between heavy alcohol intake and carotid atherosclerosis^19,20,50,51^. Given the relatively small size of the harmful drinking group in our study (N = 105), the non-significance of the markedly elevated point estimates of the differences can be first explained by a limited statistical power. Second, harmful drinkers might represent a transitional state between hazardous drinking and narcology patients, with insufficient exposure duration to manifest detectable vascular changes.

Non-drinking category demonstrated a higher atherosclerotic burden compared to non-problem drinking, with significant differences in CIMT and plaque score. These associations strengthened after adjusting for hypertension, suggesting their health condition with corresponding medication might have masked the true difference. De facto, the non-drinking group was older with higher prevalence of diabetes and statin use, suggesting their abstinence is resulting from health deficiencies rather than vice versa. An alternative explanation is that non-drinkers may lack the potential protective effects of moderate alcohol consumption. These can include improved lipid profiles^54^, lower oxidative stress and inflammation^14,15,55,56^, better endothelial function^12,13,57,58^ and insulin sensitivity^56,59^. However, the protective effects can hardly be concluded, as hazardous drinkers had broadly similar atherosclerotic manifestations to those of non-drinkers. Therefore, the reverse causation with no drinking being the consequence of health deficiencies seems the most likely explanation of the increased atherosclerotic markers in this group.

The sensitivity analysis was conducted on smaller subgroups with reduced statistical power but supported our main findings. The stratification by the presence of plaques helped address potential biases, as CIMT values are inherently higher in participants with plaques, and stenosis can only occur in the presence of plaques. Analysis of CIMT among participants without plaques showed stronger associations in narcology patients, suggesting that severe alcohol use disorder affects blood vessels before visible plaques are formed. However, when we accounted for inflammation markers, this relationship became weaker, pointing to the likely role of systemic inflammation in early vessel wall changes before plaque formation. Notably, harmful drinkers demonstrated significant CIMT increases only after the adjustment for hypertension, suggesting that in harmful drinkers, hypertension may compete with the alcohol’s direct effects on arterial wall thickness. When we looked at participants who already had plaques, the relationships between alcohol and stenosis became weaker than in our main analysis but followed the same directions. This does not necessarily mean that alcohol has less effect on the progression of atherosclerosis in its advanced stages. Rather, it likely reflects our smaller sample size in this subgroup, which made it harder to detect statistically significant differences.

The strength of our study is that we found consistent evidence linking the alcohol consumption level with atherosclerosis across multiple measures: vessel wall thickness, plaque presence, and narrowing of arteries. Nevertheless, the study has several limitations. The cross-sectional design and population-based nature do not allow concluding a causal relationship between alcohol consumption and atherosclerosis as well as to uncover the underlying pathophysiologic mechanisms. Self-reported alcohol intake may be subject to recall bias and underreporting, particularly among harmful drinkers. However, this problem was unlikely significant as our categorization was substantiated by the corresponding differences in biomarkers of alcohol exposure (CDT and GGT) in previous research on same study population^36–38^. The small sample size of harmful drinkers limited statistical power to detect specific features of this group. The findings on the non-drinking participants should be interpreted with particular caution as this group was not of primary interest in our study and likely included both lifelong abstainers and former drinkers, which could not be distinguished. Finally, our results apply only to people aged 35–69 years and may not be generalizable to populations with other drinking patterns or cardiovascular risk profiles.

From a clinical perspective, the higher values of CIMT in narcology patients (+ 0.05 mm) and hazardous drinkers (+ 0.03 mm) suggest a substantial elevation in the long-term risk for cardiovascular disasters. According to a meta-analysis by Lorenz et al., every 0.1 mm increase in CIMT is associated with a 10–15% increase in the risk of myocardial infarction and a 13–18% increase in the risk of stroke^60^. Even more critical is the doubling of the odds for hemodynamically significant carotid stenosis (OR = 2.10) in narcology patients. According to the European guidelines, a stenosis of ≥ 50% is a high-risk condition that necessitates active management, including potential intervention, to prevent ischemic stroke^61^. This means the link between excessive alcohol consumption and atherosclerosis translates into a clinically meaningful acceleration of vascular damage with implications for risk management.

## Conclusion

This study investigated the relationship between alcohol consumption levels and manifestation of carotid atherosclerosis in a Russian population sample aged 35–69 years. Narcology patients demonstrated the most severe atherosclerotic burden, with significantly increased CIMT, plaque score, and doubled odds of arterial stenosis. These associations persisted the adjustments for abdominal obesity, dyslipidemia, hypertension, diabetes, and systemic inflammation, suggesting direct alcohol-related vascular damage. Hazardous drinking showed elevated CIMT and plaque score, and these were likely mediated through hypertension. Non-drinking participants also had increased CIMT and plaque score, but this was likely reflecting other underlying health conditions rather than alcohol abstinence effects.

## Supplementary Information

Below is the link to the electronic supplementary material.

Supplementary Material 1

## Data Availability

Data from the Know Your Heart Study are available from the authors upon reasonable request with permission of the Know Your Heart Steering Group (https://metadata.knowyourheart.science/).
