# Supplementary material for: Association between alcohol consumption and carotid atherosclerosis in Russia
Source: Sci Rep. 2025 Aug 27;15:31646. doi: 10.1038/s41598-025-16290-0 (PMC12391389; doi:10.1038/s41598-025-16290-0)
Supplement: Supplementary file 1 — Supplementary Material 1 [file 41598_2025_16290_MOESM1_ESM.docx]

**Supplementary Materials**

**Supplementary Table 1.** Regression models for associations between alcohol consumption levels and intima media thickness in participants without plaques (N=1,023) and for the presence of stenosis in participants with plaques (N=1,606)

| Outcomes and Drinking Groups | Model 1 | Model 2a | Model 2b | Model 2c | Model 2d | Model 2e | Model 3 |
| --- | --- | --- | --- | --- | --- | --- | --- |
| Carotid Intima Media Thickness (mm), quantile regressions, B (95%CI) | | | | | | | |
| Non-drinking (N=68, 28.8%) | -0.01 (-0.04; 0.03) | 0.01 (-0.02; 0.04) | -0.01 (-0.04; 0.02) | 0.01 (-0.02; 0.05) | -0.01 (-0.04; 0.03) | -0.00 (-0.04; 0.03) | 0.01 (-0.02; 0.04) |
| Non-problem (N=728, 44.0%) | Ref | Ref | Ref | Ref | Ref | Ref | Ref |
| Hazardous  (N=122, 33.9%) | **0.03 (0.00; 0.06)** | 0.03 (-0.00; 0.06) | **0.04 (0.01; 0.06)** | 0.03 (-0.00; 0.06) | 0.02 (-0.01; 0.05) | 0.02 (-0.01; 0.05) | 0.03 (-0.00; 0.07) |
| Harmful (N=25, 23.8%) | 0.01 (-0.03; 0.05) | 0.02 (-0.00; 0.04) | 0.01 (-0.03; 0.06) | **0.02 (0.00; 0.03)** | -0.00 (-0.05; 0.04) | 0.02 (-0.04; 0.09) | 0.02 (-0.01; 0.05) |
| Narcology (N=80, 29.4%) | **0.04 (0.00; 0.07)** | **0.06 (0.03; 0.09)** | **0.05 (0.02; 0.08)** | **0.05 (0.03; 0.08)** | **0.04 (0.00; 0.07)** | 0.03 (-0.00; 0.07) | **0.06 (0.04; 0.09)** |
| Presence of Stenosis, logistic regressions, OR (95%CI) | | | | | | | |
| Non-drinking (N=168, 71.2%) | 1.34 (0.82; 2.20) | 1.33 (0.81; 2.20) | 1.35 (0.82; 2.22) | 1.38 (0.84; 2.26) | 1.34 (0.82; 2.21) | 1.36 (0.83; 2.24) | 1.39 (0.85; 2.30) |
| Non-problem (N=928, 56.0%) | Ref | Ref | Ref | Ref | Ref | Ref | Ref |
| Hazardous (N=238, 66.1%) | 1.11 (0.69; 1.77) | 1.12 (0.70; 1.79) | 1.11 (0.69; 1.77) | 1.03 (0.64; 1.67) | 1.11 (0.69; 1.78) | 1.10 (0.69; 1.77) | 1.06 (0.65; 1.71) |
| Harmful (N=80, 76.2%) | 1.66 (0.87; 3.17) | 1.65 (0.86; 3.16) | 1.67 (0.88; 3.19) | 1.66 (0.88; 3.12) | 1.67 (0.88; 3.18) | 1.67 (0.88; 3.17) | 1.64 (0.87; 3.11) |
| Narcology (N=192, 70.6%) | 1.46 (0.82; 2.57) | 1.43 (0.80; 2.54) | 1.53 (0.87; 2.70) | 1.67 (0.95; 2.95) | 1.48 (0.84; 2.62) | 1.46 (0.83; 2.57) | 1.65 (0.93; 2.94) |
| Significant associations (p<0.05) are highlighted in bold. Model 1: adjusted for sex, age, higher education, marital status, smoking; Model 2a: adjusted as Model 1 plus abdominal obesity; Model 2b: adjusted as Model 1 plus dyslipidemia; Model 2c: adjusted as Model 1 plus hypertension; Model 2d: adjusted as Model 1 plus diabetes; Model 2e: adjusted as Model 1 plus systemic inflammation; Model 3: adjusted as Model 1 plus abdominal obesity, dyslipidemia, hypertension, diabetes, systemic inflammation. | | | | | | | |

**Supplementary Table 2.** Fully adjusted regression models for associations of alcohol consumption levels and sociodemographic and dysmetabolic characteristics with carotid atherosclerosis markers (Model 3)

| Predictor Variable | CIMT (mm)¹ | Plaque score (points)² | Presence of stenosis³ |
| --- | --- | --- | --- |
|  | B (95% CI) | B (95% CI) | OR (95% CI) |
| Alcohol Consumption Levels |  |  |  |
| Non-problem drinking | Ref. | Ref. | Ref. |
| Non-drinking | 0.02 (0.00, 0.04) | 0.30 (0.11, 0.49) | 1.54 (0.93, 2.54) |
| Hazardous drinking | 0.03 (0.01, 0.05) | 0.15 (-0.01, 0.31) | 1.14 (0.71, 1.84) |
| Harmful drinking | -0.00 (-0.03, 0.03) | 0.20 (-0.09, 0.49) | 1.85 (0.98, 3.48) |
| Narcology patients | **0.05 (0.03, 0.08)** | **0.57 (0.35, 0.79)** | **2.10 (1.18, 3.76)** |
| Sociodemographic factors |  |  |  |
| Sex (Male vs. Female) | **-0.05 (-0.07, -0.04)** | **-0.57 (-0.69, -0.46)** | **0.57 (0.39, 0.84)** |
| Age (years) | **0.01 (0.01, 0.01)** | **0.07 (0.06, 0.08)** | **1.10 (1.08, 1.13)** |
| Higher education | 0.00 (-0.01, 0.02) | -0.03 (-0.14, 0.08) | 0.81 (0.56, 1.17) |
| Married | 0.00 (-0.01, 0.01) | -0.04 (-0.14, 0.07) | 1.21 (0.85, 1.73) |
| Current smoker | **0.03 (0.02, 0.05)** | **0.52 (0.39, 0.66)** | **2.77 (1.92, 3.99)** |
| Dysmetabolic Conditions |  |  |  |
| Abdominal obesity | **0.03 (0.02, 0.04)** | -0.09 (-0.21, 0.03) | 0.79 (0.53, 1.16) |
| Dyslipidemia | **0.02 (0.01, 0.04)** | **0.24 (0.11, 0.37)** | 1.38 (0.85, 2.23) |
| Hypertension | **0.04 (0.03, 0.06)** | **0.38 (0.27, 0.49)** | **2.54 (1.65, 3.90)** |
| Diabetes | 0.02 (-0.00, 0.05) | 0.14 (-0.06, 0.34) | 1.23 (0.79, 1.93) |
| Systemic inflammation | 0.01 (-0.01, 0.02) | 0.05 (-0.06, 0.15) | 1.08 (0.79, 1.48) |
| Statistically significant associations (p<0.05) are highlighted in bold. All models are adjusted for all variables listed in the table.  ¹ Quantile regression for carotid intima-media thickness (CIMT).  ² Linear regression for plaque score.  ³ Logistic regression for the presence of stenosis ≥50%. | | | |
